# TRAF2 binds to TIFA via a novel motif and contributes to its autophagic degradation

**DOI:** 10.1002/1873-3468.70110

**Published:** 2025-07-22

**Authors:** Tom Snelling, Kodi Hunter, Celest W. S. Tay, Nicola T. Wood, Philip Cohen

**Affiliations:** ^1^ MRC Protein Phosphorylation and Ubiquitylation Unit, School of Life Sciences University of Dundee UK; ^2^ Present address: Wellcome Sanger Institute, Wellcome Genome Campus Cambridge England UK

**Keywords:** alphafold3, ALPK1, Autophagy, TIFA, TRAF2

## Abstract

A signal transduction pathway has been defined in which ADP‐heptose activates the mammalian protein kinase ALPK1, which phosphorylates the adaptor protein TIFA, inducing its polymerisation and interaction with the E3 ubiquitin ligases TRAF2/c‐IAP1 and TRAF6. These E3 ligases drive activation of the transcription factors NF‐κB and AP‐1, culminating in the production and secretion of inflammatory mediators to combat microbial infection. TRAF6 is essential in this process, but how TRAF2 interacts with TIFA and its role in the pathway is unclear. Here, we identify two conserved sequence motifs in TIFA essential for TRAF2 interaction, one of which (Pro159‐Xaa‐Xaa‐Glu162) is novel. We additionally report that ADP‐heptose induces TIFA degradation by autophagy and that both TRAF2 and TRAF6 contribute to this process. These findings advance understanding of how TRAF2 regulates the ALPK1–TIFA signalling pathway.

## Graphical abstract

TRAF family members couple receptor signalling complexes to downstream outputs, but how they interact with these complexes is not always clear. Here, we show that during ADP‐heptose signalling, TRAF2 binding to TIFA requires two short sequence motifs in the C‐terminal tail of TIFA, which are distinct from the TRAF6 binding motif.

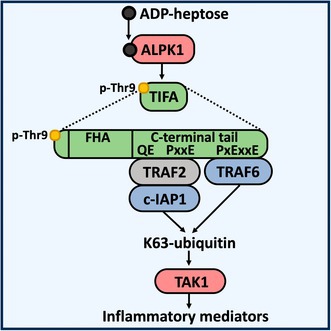

## Abbreviations

**ALPK1**, alpha‐kinase 1

**c‐IAP1**, cellular inhibitor of apoptosis protein‐1

**FHA**, forkhead‐associated

**GAPDH**, glyceraldehyde 3‐phosphate dehydrogenase

**IKK**, IκB kinase

**IκB**, inhibitor of κB

**KO**, knockout

**NF‐κB**, nuclear factor‐κB

**TAK1**, TGFβ‐activated kinase 1

**TBK1**, TANK‐binding kinase 1

**TIFA**, TRAF‐interacting and FHA domain‐containing protein

**TRAF**, TNF‐receptor‐associated factor

**WT**, wild‐type

Over 20 years ago, a protein termed TRAF2‐binding protein was reported to activate the transcription factors NF‐κB and AP‐1 when overexpressed in HEK293 cells [1]. Subsequently, it was observed to activate the canonical IκB kinase (IKK) complex (which activates NF‐κB) in an *in vitro* reconstitution system that required the E3 ubiquitin ligase TRAF6 and the protein kinase TAK1 and was renamed TRAF‐Interacting and Forkhead‐associated (FHA) domain‐containing protein (TIFA) [2]. Other investigators later reported that the overexpression of TIFA in HEK293 cells resulted in its phosphorylation at Thr9, causing TIFA to polymerise and interact with TRAF6 [3]. The polymerisation of TIFA was dependent on Thr9 and the FHA domain of TIFA, suggesting that TIFA polymerises via the interaction of phospho‐Thr9 of one TIFA molecule with the FHA domain of another to produce polymeric forms of the protein, which have been termed TIFAsomes [3, 4, 5].

Genetic screens performed to identify genes required for the TIFA‐ and NF‐κB‐dependent expression of IL‐8 in HeLa cells [4] or the nuclear translocation of the p65 subunit of NF‐κB in AGS cells [5] identified the atypical protein kinase alpha‐kinase 1 (ALPK1), as well as TRAF6 and TAK1, as essential components of a signalling pathway triggered by exposure to bacterial pathogens. These investigators additionally showed that TIFA formed oligomers in infected cells, which could be prevented by the depletion of ALPK1 or by the mutation of Thr9 in TIFA to Ala [4, 5]. Subsequently, ALPK1 was found to phosphorylate TIFA at Thr9 in human cells and to be activated allosterically by ADP‐heptose [6], a bacterial nucleotide sugar required for the incorporation of heptose into the inner and outer cores of lipopolysaccharide [7] and as a constituent of S‐layer glycoproteins present on the cell surface of bacteria and archaea [8]. Recently, ALPK1 was also shown to be activated by other microbial nucleotide‐heptoses (UDP‐heptose and CDP‐heptose) [9], which are produced by the same class of nucleotidyltransferases that synthesise ADP‐heptose. These molecules are made by some bacteria, viruses, fungi and archaea, but are not known to be produced by vertebrates. Taken together, these findings suggest that ALPK1 mediates a cross‐kingdom immune surveillance mechanism that protects humans from a range of pathogens [9].

Both TRAF2 and TRAF6 contain a RING domain, which commonly exhibit E3 ligase activity. However, despite their structural similarity, only TRAF6 functions as an E3 ligase. TRAF2, in contrast, is not an active enzyme and instead forms a complex with the E3 ligase c‐IAP1 [10]. The TIFAsomes formed in response to ADP‐heptose recruit TRAF6 and TRAF2/c‐IAP1, which in turn produce the Lys63‐linked ubiquitin chains that are required to activate the TAK1 protein kinase complex [11, 12]. TAK1 then activates the canonical IKK complex and c‐Jun N‐terminal kinases, which switch on the transcription factors NF‐κB and AP‐1 (activator protein 1), respectively. Importantly, the ADP‐heptose‐dependent activation of the canonical IKK complex and c‐Jun N‐terminal kinases is abolished in TRAF6 knockout (KO) cells, but not in cells expressing an E3 ligase‐inactive mutant of TRAF6, because under these conditions, c‐IAP1 can generate the Lys63‐linked ubiquitin chains that are required to activate TAK1 [12]. These observations also revealed that the TRAF6 protein has an essential, but unknown, function in ADP‐heptose signalling, which is independent of its E3 ligase activity [12].

TRAF6 binds to proteins containing a Pro‐Xaa‐Glu‐Xaa‐Xaa‐Zaa motif (where Zaa is an aromatic or acidic amino acid residue and Xaa can be any amino acid residue) [13]. TIFA contains such a motif (Pro‐Thr‐Glu‐Met‐Asp‐Glu) located between amino acid residues 176 and 181, close to its C‐Cterminus. A TIFA mutant in which Glu178 was changed to Ala failed to activate the canonical IKK complex in an *in vitro* reconstitution system [2], and the same mutation abolished the ADP‐heptose‐dependent interaction of TRAF6 with TIFA in HEK293 cells [12]. In contrast, the ADP‐heptose‐dependent interaction between TRAF2 and TIFA was unimpaired by the mutation of Glu178 to Ala in TIFA [12], indicating that the binding sites for TRAF2 and TRAF6 on TIFA are distinct. However, the specific amino acid residues required for the interaction of TIFA with TRAF2 are unknown. TRAF2 has important roles in signalling by tumour necrosis factor (TNF) and other TNF superfamily members, and two distinct TRAF2‐interacting motifs have been identified, namely (Pro/Ser/Ala/Thr)‐Xaa‐(Gln/Glu)‐Glu and (Pro‐Xaa‐Gln‐Xaa‐Xaa‐Asp) [14]. However, our inspection of the amino acid sequence of TIFA failed to recognise either motif, raising the question of how TIFA interacts with TRAF2. Here, we identify the residues in TIFA that interact with TRAF2. We also show that ADP‐heptose induces the degradation of TIFA by an autophagic mechanism that requires the expression of TRAF2 or TRAF6.

## Material and Methods

### Structural prediction

The amino acid sequences of human TIFA (Q96CG3) and human TRAF2 (Q12933) were obtained from the uniprot database, and their three‐dimensional structures were predicted using the alphafold3 algorithm with default settings (https://alphafoldserver.com/). The structural models were downloaded and analysed using pymol (v3.1) to investigate the spatial arrangement of amino acid residues.

### DNA constructs

The following DNA plasmids encoding FLAG‐tagged TIFA or TIFA mutants with expression under the control of the UbC promoter were made by Medical Research Council Reagents and Services, Medical Research Council Protein Phosphorylation and Ubiquitylation Unit, University of Dundee and assigned unique identifiers (available by request from https://mrcppureagents.dundee.ac.uk/): WT TIFA (DU71017), TIFA[1–172] (DU79168), TIFA[1–165] (DU79189), TIFA[1–157] (DU79169), E151A (DU79184), E151Q (DU79200), E151D (DU79202), E162A (DU79129), E162D (DU80383), E162Q (DU80336), P159A (DU79175), P159Q (DU78475), P161E (DU79177), P161Q (DU78468), P159D (DU79201) and P159S (DU79210).

### Antibodies

The following antibodies were obtained from Cell Signalling Technology (CST) (Danvers, MA, USA): a phospho‐specific antibody recognising NF‐κB1 phosphorylated at Ser932 (#4806) and antibodies recognising anti‐rabbit IgG (#7074) and anti‐mouse IgG (#7076), GAPDH (#2118), c‐IAP1 (#7065), IκBα (#4812), TRAF2 (#4712) and TBK1 (#3013). Antibodies recognising TIFA (#ab239352) and TRAF6 (#ab40675) were from Abcam (Cambridge, UK).

### Agonists and inhibitors

ADP‐heptose (#tlrl‐adph‐l), BV‐6 (#inh‐bv6), chloroquine (#tlrl‐chq‐4) and bafilomycin A1 (#tlrl‐baf1) were purchased from Invivogen (San Diego, CA, USA), while E64‐D (#E8640) and bortezomib (#5043140001) were from Sigma‐Aldrich (St. Louis, MO, USA). ADP‐heptose (10 mm), BV‐6 (10 mm), E64‐D (1 mg·mL^−1^), bortezomib (20 mm), bafilomycin A1 (50 μm) and chloroquine (50 mm) were dissolved in either DMSO (BV‐6, E64‐D, bortezomib and bafilomycin A1) or PBS (ADP‐heptose and chloroquine) to generate stock solutions at the concentrations indicated in parentheses. These stock solutions were then added to culture media for the times and at the final concentrations indicated in figure legends.

### Cell culture, cell maintenance and cell lysis

TIFA knockout HEK‐Blue cells (#hkb‐kotifa; CVCL_A8BV) and their parental cell line (#hkb‐null1v; CVCL_A7YL) were purchased from Invivogen in the past 3 years and were not authenticated, while the wild‐type, TRAF6 and TRAF2/TRAF6 double knockout HEK293 cells (CVCL_0030) used have been described previously [12] and were authenticated for this study through STR profiling (Eurofins, Luxembourg). In the present study, TRAF2 and TBK1 knockout HEK‐Blue cells were generated from their parental cell line (CVCL_A7YL) using a protocol described previously [12]. The guide sequences for TRAF2 were GACCCTCCTGGGGACCAAGC and GCCGGGCTGTAGCAACTCCA, and for TBK1 were GATGCGTGTTATAGGGGAAGA and GTATTTCCTGGCTTGATATCA. All cell lines were cultured at 37 °C in a humidified atmosphere with 5% CO_2_, and the growth medium was Dulbecco's modified Eagle's medium (DMEM) supplemented with 10% (v/v) foetal bovine serum (FBS), 2 mm L‐glutamine, 100 U·mL^−1^ penicillin and 0.1 mg·mL^−1^ streptomycin and were regularly confirmed to be free of mycoplasma contamination.

To prepare cell lysates, the cells were washed once with ice‐cold PBS and scraped from the plate into lysis buffer (50 mm Tris/HCl (pH 7.5), 1 mm EDTA, 1 mm EGTA, 1% (v/v) Triton X‐100, 270 mm sucrose, Halt protease and phosphatase inhibitor cocktail (#78441; ThermoFisher) and 1 mm DTT). The subsequent processing of the cell extracts varied depending on whether the lysates were to be used to analyse the binding of TIFA to TRAF2 or to study TIFA degradation. For the former, cell lysates were clarified by centrifugation for 20 min at 20 000 ***g*** at 4 °C and the supernatant (cell extract) subjected to either immunoprecipitation or analysed by SDS/PAGE directly following the addition of lithium dodecyl sulphate (LDS) sample buffer (#NP0007; ThermoFisher, Waltham, MA, USA) supplemented with 2.5% (v/v) *β*‐mercaptoethanol. For experiments assessing TIFA degradation, the lysates were not clarified. Instead, LDS sample buffer containing 2.5% (v/v) *β*‐mercaptoethanol was added directly to the cell lysates, heated to 95 °C for 5 min and probe‐sonicated for 10 cycles (30 s on, 30 s off, 70% amplitude) and centrifuged for 2 min at 20 000 ***g***.

### Transient transfection of HEK293 cells and co‐immunoprecipitation from cell extracts

The method used to transfect TIFA KO cells with FLAG‐tagged TIFA constructs and analyse the interaction of the overexpressed TIFA with TRAF2 by co‐immunoprecipitation has been described in detail elsewhere (https://doi.org/10.17504/protocols.io.x54v926xml3e/v1). Briefly, TIFA knockout HEK‐Blue cells were ‘reverse’ transfected with plasmid DNA using lipofectamine 2000 (#11668027; Invitrogen, Paisley, UK), according to the manufacturer's protocol. After 24 h, cells were lysed and clarified as described in the previous section. Ten percent of the resulting cell extract by volume was used directly for SDS/PAGE and immunoblotting, and the remaining 90% was subjected to immunoprecipitation for 16 h at 4 °C on a rotating wheel using 20 μL anti‐FLAG M2 affinity gel (#A2220; Sigma‐Aldrich), which had been pre‐washed three times with lysis buffer. After centrifugation, the supernatant was discarded, and the pelleted affinity gel was washed three times with wash buffer (50 mm Tris/HCl (pH 7.5), 1% (v/v) Triton X‐100 and 1 mm DTT) supplemented with 250 mm NaCl and twice with wash buffer. The immunoprecipitates were then denatured in LDS sample buffer and incubated for 5 min at 30 °C on a thermomixer (1200 r.p.m.) to elute the bound proteins. The supernatant was collected, supplemented with *β*‐mercaptoethanol to a final concentration of 2.5% (v/v), and the sample was heated to 95 °C for 5 min prior to analysis by SDS/PAGE.

### SDS/PAGE, transfer to PVDF membranes and immunoblotting

Cell extracts or immunoprecipitates were resolved by SDS/PAGE, transferred to PVDF membranes and incubated for 1 h in 50 mm Tris/HCl pH 7.5, 150 mm NaCl and 0.1% (v/v) Tween‐20 (TBS‐T) containing 5% (w/v) non‐fat milk powder (Marvel, Premier Foods, St Albans, UK). Primary antibody in TBS‐T containing 5% (w/v) bovine serum albumin was then incubated with the PVDF membrane on a shaker for 16 h at 4 °C. The immunoblots were washed five times (10 min per wash) with TBS‐T, followed by incubation for 1 h with the appropriate horseradish peroxidase‐conjugated secondary antibody in TBS‐T containing 5% (w/v) non‐fat milk powder and washed a further five times with TBS‐T. Blots were developed by incubation at ambient temperature with Amersham ECL Select Western Blotting Detection Reagent (GE Healthcare Ltd, Chalfont St. Giles, UK) or Super‐Signal West Pico Chemiluminescent Substrate (Merck‐Millipore, Darmstadt, Germany) and imaged using a ChemiDoc MP scanner (#17001402; Bio‐Rad, Hercules, CA, USA).

## Results

### The structure of a TIFA oligomer predicted by alphafold3

**Fig. 1:**
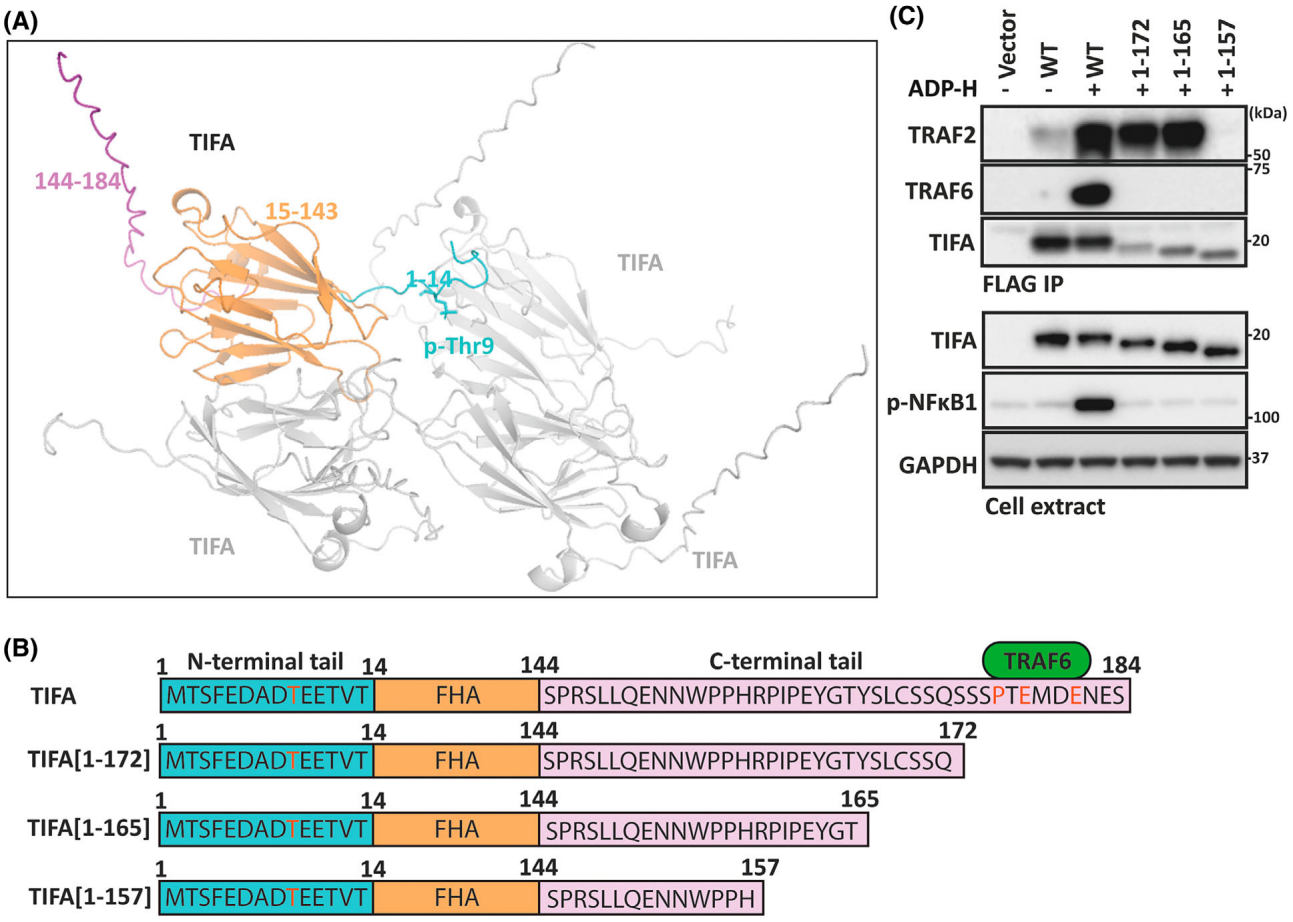
Amino acid residues 158–165 in TIFA are essential for its ADP‐heptose‐dependent interaction with TRAF2. (A) alphafold3 model of a tetramer of full‐length wild‐type human TIFA with Thr9 phosphorylated and one TIFA molecule highlighted. The disordered *N*‐terminal region (residues 1–14) is in blue, with phosphorylated Thr9 (pThr9) shown in stick representation and labelled. The structured region, which includes the FHA domain (residues 15–143), is in orange. The disordered *C*‐terminal tail (residues 144–184) is in pink. The other three TIFA molecules are shown in grey. (B) Schematic representation of full‐length and truncated TIFA constructs coloured as in (A). The amino acid sequences of the disordered *N*‐ and *C*‐terminal regions are shown, with Thr9 in the *N*‐terminal region and the TRAF6 binding motif in the *C*‐terminal region highlighted in red. TRAF6 itself is highlighted in green. The truncated versions of TIFA tested for TRAF2 and TRAF6 binding in (C) are also shown. (C) TIFA knockout HEK‐blue cells were transfected with an empty vector or plasmids encoding full‐length FLAG‐tagged TIFA or the FLAG‐tagged TIFA truncation mutants shown in (B). After 24 h, cells were stimulated for 20 min with or without 10 μm ADP‐heptose and lysed. FLAG immunoprecipitation (IP) was performed on the lysates, followed by SDS/PAGE and immunoblotting with the antibodies indicated (upper section, labelled FLAG IP). Cell lysates were also analysed by SDS/PAGE and immunoblotting with the antibodies indicated (bottom section, labelled cell extract). The immunoblots are representative of two independent experiments.

Although the three‐dimensional structure of full‐length, wild‐type (WT) human TIFA has not yet been elucidated experimentally, the structure of TIFA containing the Thr9Asp and Cys36Ser mutations [15] and a truncated form of TIFA lacking the disordered *C*‐terminal region [16] has been solved by X‐ray crystallography. However, these TIFA mutants are unable to mediate ADP‐heptose signalling in cells, because the Thr9Asp mutant does not mimic the effect of phosphorylation and the *C*‐terminal truncation removes the TRAF6 binding site [12]. Recent advances in computational biology have transformed the ability to predict protein structures, with alphafold3 leading the way [17]. We therefore used alphafold3 to predict the structure of full‐length WT TIFA in which Thr9 was phosphorylated. This model predicted a structure in which TIFA intrinsic dimers interact in an anti‐parallel fashion, showing how they interact in a phosphorylation‐dependent manner (Fig. 1A). We did not focus on analysing the exact number of dimers that come together to form the oligomeric state, which has been suggested to be hexameric in one of the published structures [15]. Importantly, our model predicted an ordered structure from amino acid residues 15–143 of TIFA that included the FHA domain, while the *N*‐terminal 14 amino acid residues (1–14) and the *C*‐terminal 40 amino acid residues (144–184) were disordered. As expected, alphafold3 predicted that phospho‐Thr9 interacted with the FHA domain of another TIFA molecule (Fig. 1A) and explained, at least in part, how phosphorylation may induce the oligomerisation of TIFA.

### The *C*‐terminal truncation of TIFA leads to the sequential loss of TRAF6 and TRAF2 binding

Since TRAF6 binds to the disordered C‐terminal tail of TIFA through the Pro‐Xaa‐Glu‐Xaa‐Xaa‐Glu motif between amino acid residues 176 and 181 [2], we wondered whether the same disordered region, spanning amino acid residues 144–184, might also bind to TRAF2. We tested this hypothesis by progressively truncating the C‐terminus of TIFA as depicted schematically in Fig. 1B. We found by co‐immunoprecipitation that TIFA[1–172] lacking the *C*‐terminal 12 amino acid residues of TIFA and hence the TRAF6 binding motif did not interact with TRAF6 as expected (Fig. 1B and C) and hence also abolished the phosphorylation of NF‐κB1, a substrate of IKKβ, which is critically dependent on the expression of TRAF6 (Fig. 1C) [12]. However, the binding of TIFA to TRAF2 was unaffected by this truncation, confirming that the TRAF2 and TRAF6 binding sites are distinct. The binding of TRAF2 to TIFA was also unaffected in the TIFA[1–165] truncation mutant but undetectable in the TIFA[1–157] mutant, indicating that one or more of the amino acid residues in the sequence RPIPEYGT between 158 and 165 might be important for TRAF2 binding (Fig. 1B and C).

### Pro159 and Glu162 of TIFA are required for interaction with TRAF2

**Fig. 2:**
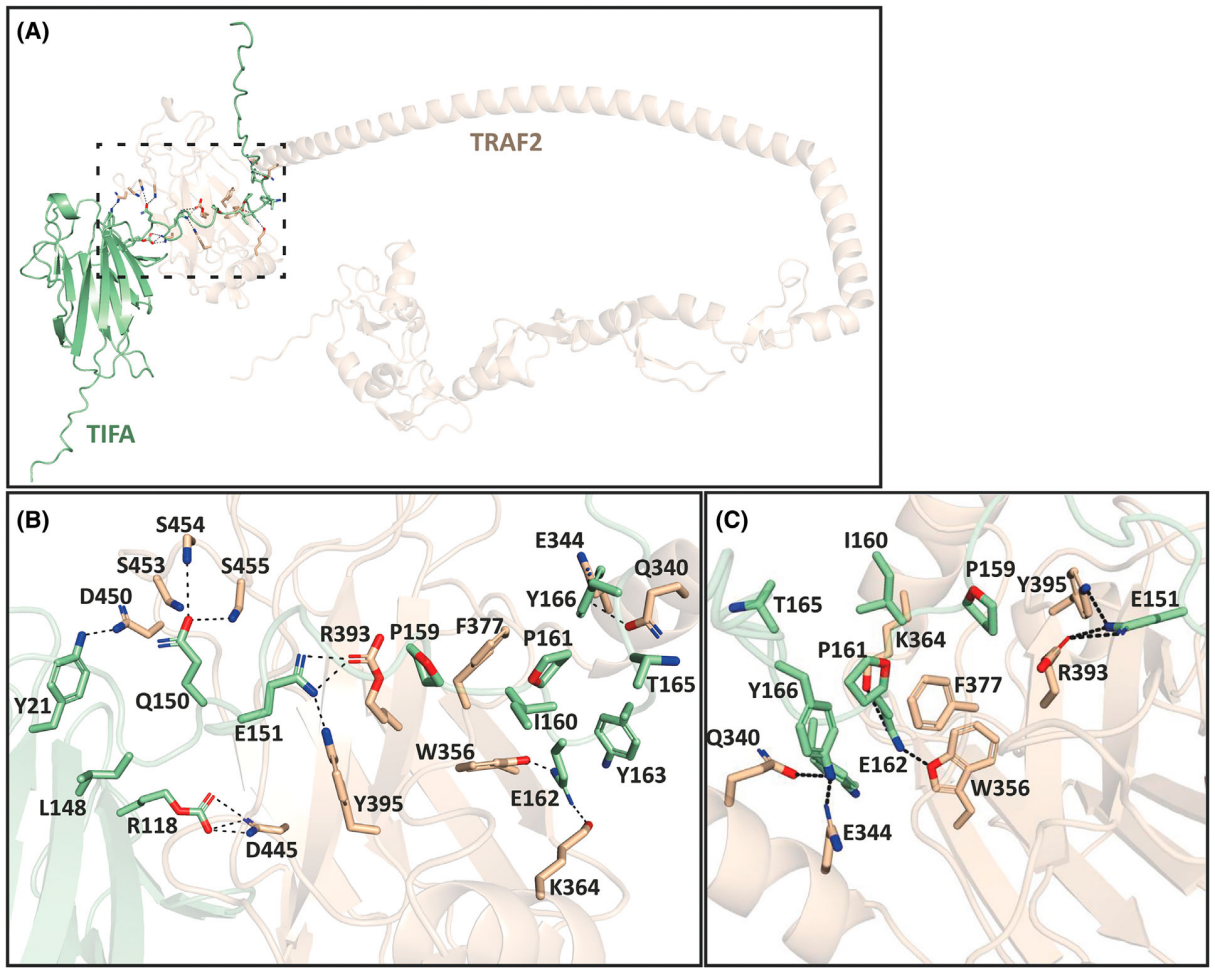
Model of the interaction of full‐length wild‐type TIFA with the TRAF‐C domain of TRAF2. (A) alphafold3 model showing the predicted interaction between full‐length TIFA and full‐length TRAF2. The dotted panel shows the key interacting region. (B) Close‐up of the rectangular area depicted by the broken lines in (A) showing the predicted interactions between TIFA and TRAF2. (C) Similar to (B), but the viewpoint is rotated to show the interactions from a different viewing angle.

**Fig. 3:**
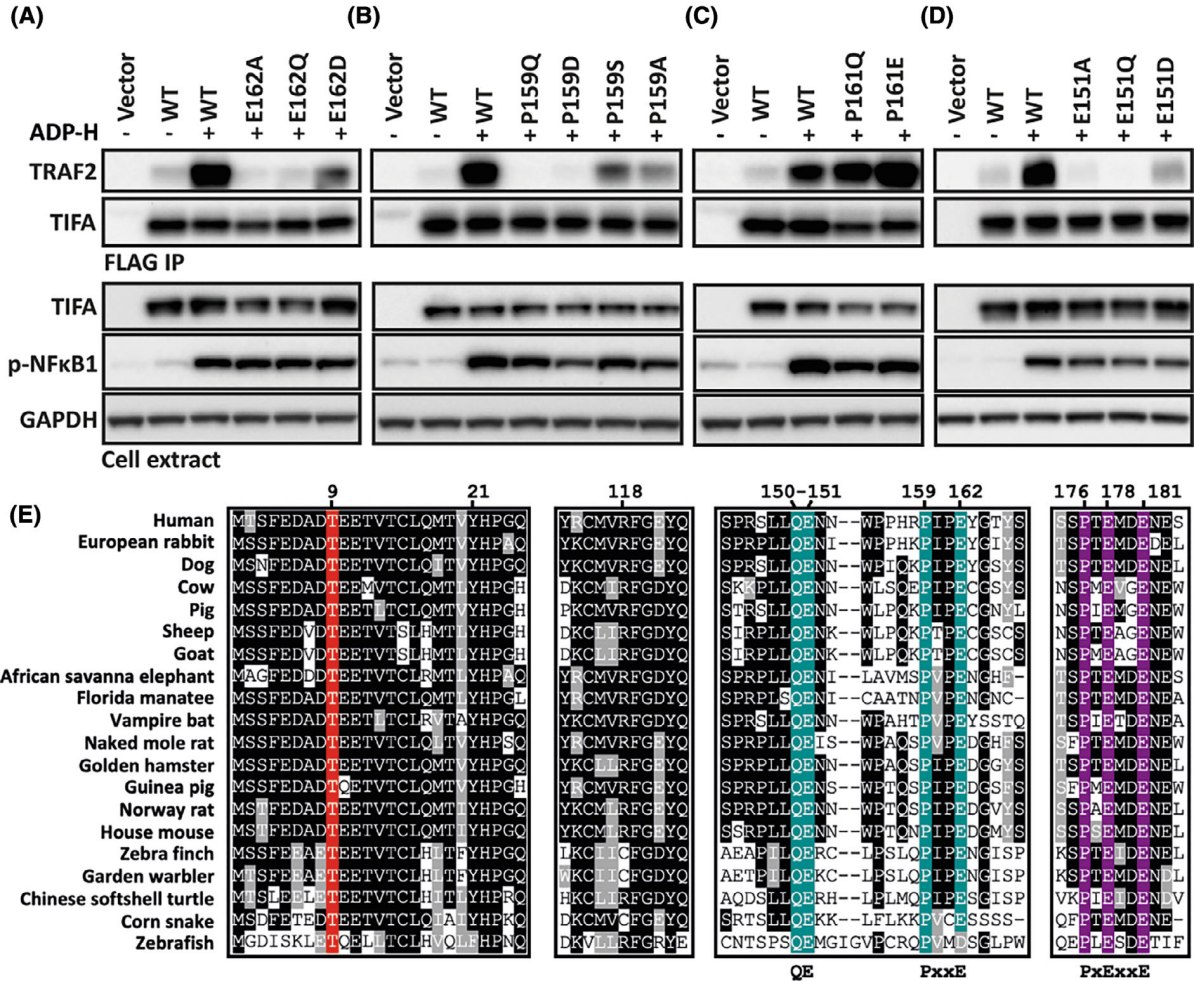
Conservation of the TRAF2 and TRAF6 binding residues in TIFA during vertebrate evolution (A) TIFA knockout HEK‐blue cells were transfected with empty vector or plasmid DNA encoding FLAG‐tagged wild‐type TIFA or the indicated TIFA mutants. After 24 h, cells were stimulated for 20 min with or without 10 μM ADP‐heptose and lysed. FLAG was immunoprecipitated from the lysates, followed by SDS/PAGE and immunoblotting as in Fig. 1C (upper panel, labelled FLAG IP). Cell lysates were also analysed as in Fig. 1C (lower panel, labelled cell extract). (B–D) As in (A), except that different TIFA mutants were used. (E) Multiple sequence alignment of a subset of TIFA residues from the indicated vertebrates with numbering relative to human TIFA. Conservation of the key amino acid residues that interact with TRAF2 and TRAF6 is highlighted on turquoise and purple backgrounds, respectively, and the residue phosphorylated by ALPK1 (Thr9) on a red background. Conserved motifs discussed in this manuscript are shown below the alignment, where x represents any amino acid residue. Immunoblots shown in (A–D) are representative of at least two independent experiments.

We next used alphafold3 to predict how full‐length TIFA would interact with the TRAF‐C domain of TRAF2 (amino acid residues 264–501), which is known to mediate the binding of TRAF2 to other TRAF2‐interacting proteins [14]. The model suggested that Glu162 of TIFA interacts with Trp356 and Lys364 in TRAF2, while Pro159 is located close to Arg393 (Fig. 2A–C). In contrast, Ile160, Tyr163, Gly164, or Thr165 of TIFA face away from TRAF2 in the alphafold3 model and, therefore, do not interact with TRAF2 (Fig. 2A–C). We therefore tested the model by mutating Pro159 and Glu162 to other amino acid residues. We found that the binding of TRAF2 to TIFA was abolished by the mutation of Glu162 to Ala or Gln and reduced by its mutation to Asp (Fig. 3A), indicating that an acidic amino acid residue at this position (especially Glu) in TIFA is critical for binding to TRAF2. The model also placed Pro159 in a position such that its mutation to an amino acid with a bulky or charged side chain would cause steric hindrance or disrupt interactions with Arg393 of TRAF2. Consistent with the model, we found that the binding of TRAF2 to TIFA was abolished by the mutation of Pro159 to either Gln or Asp and reduced considerably by its mutation to Ser or Ala (Fig. 3B). Since Pro159 and Glu162 are located within the linear sequence Pro159‐Ile160‐Pro161‐Glu162, we wondered whether Pro161 was also important. However, the mutation of this residue to Gln or Glu did not reduce interaction with TRAF2 (Fig. 3C). The model also predicted that Tyr166 of TIFA interacts with Glu344 and Gln340 of TRAF2, but since the TIFA[1–165] truncation mutant lacking Tyr166 bound TRAF2 similarly to WT TIFA (Fig. 1), these interactions cannot be critical for TRAF2 binding.

Taken together, these experiments indicated that Pro159 and Glu162 are the critical amino acid residues for TRAF2 binding within the amino acid sequence RPIPEYGT, with Glu162 forming electrostatic interactions with Trp356 and Lys364 of TRAF2 (Fig. 2A–C). This is consistent with the conservation of Pro159 and Glu162 throughout vertebrate evolution, whereas Pro161 and Tyr166 are poorly conserved (Fig. 3E). The exception is zebrafish, in which Glu162 is replaced by Asp (Fig. 3E) and we found that when Glu162 was replaced by Asp in human TIFA, this mutation reduced but did not abolish the binding of TIFA to TRAF2 (Fig. 3A). In contrast to TRAF2 and TRAF6, neither TIFA nor ALPK1 are found within invertebrate lineages.

### Gln150 and Glu151 of TIFA are required for ADP‐heptose‐dependent interaction with TRAF2

Importantly, the alphafold3 model also predicted that amino acid residues Gln150 and Glu151 of TIFA interact with TRAF2. These two residues lie within the sequence Leu‐Leu‐Gln‐Glu, which resembles the canonical TRAF2 binding motif (Pro/Ser/Ala/Thr)‐Xaa‐(Glu/Gln)‐Glu found in all TNF receptor superfamily members, except that the first amino acid is Leu. Moreover, as found in other TRAF2 binding proteins, alphafold3 predicted that Gln150 of TIFA interacted with Ser454 and Ser455 of TRAF2 while Glu151 interacted with Arg393 and Tyr395 (Fig. 2). The importance of Glu151 was confirmed by the finding that its mutation to Gln or Ala abolished binding to TRAF2, while its mutation to Asp reduced binding (Fig. 3D). The importance of Gln150 and Glu151 was also indicated by their conservation throughout vertebrate evolution (Fig. 3E).

Although the mutation of Glu151 to Ala or Gln, the mutation of Pro159 to Asp or Gln, or the mutation of Glu162 to Ala or Gln abolished the interaction of TIFA with TRAF2, they had little to no effect on the ADP‐heptose‐stimulated phosphorylation of NF‐κB1 (Fig. 3), indicating that the interaction of TIFA with TRAF6, which is required for NF‐κB1 phosphorylation [12], remained intact.

### ADP‐heptose signalling induces the degradation of TIFA by autophagy

**Fig. 4:**
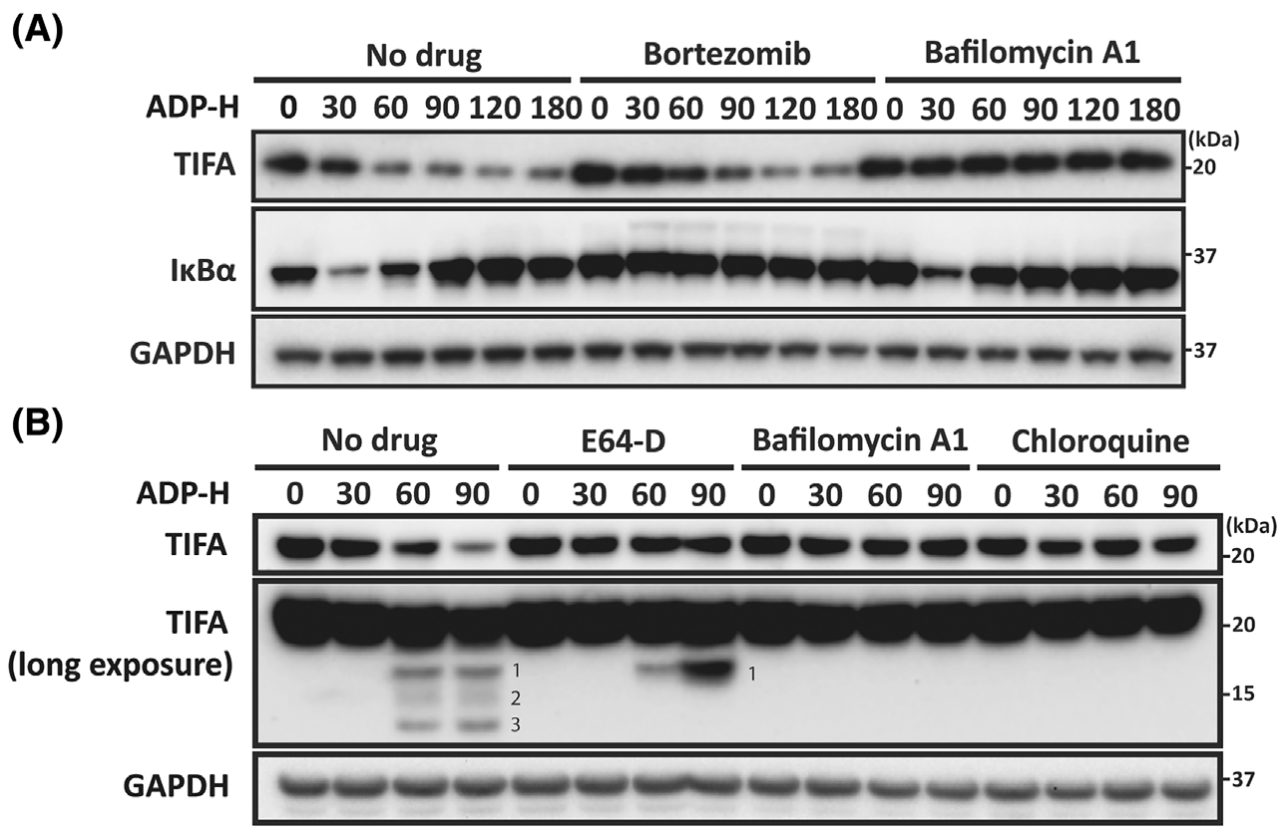
ADP‐heptose stimulates the degradation of TIFA by an autophagy‐dependent pathway. (A) Wild‐type HEK‐blue cells were incubated for 2 h in the absence (no drug) or presence of bortezomib (20 μm) or bafilomycin A1 (500 nm), then stimulated for the times indicated with 30 μm ADP‐heptose (ADP‐H) followed by cell lysis (see Methods), SDS/PAGE and immunoblotting with the antibodies indicated. (B) As in (A), except that the cells were incubated with E64‐D (1.0 μg·mL^−1^), bafilomycin A1 (500 nm) or chloroquine (0.1 mm) prior to stimulation with ADP‐heptose. Extended exposure of a TIFA immunoblot revealed small amounts of faster migrating species (labelled 1, 2 and 3), which are likely to be formed in lysosomes. The immunoblots shown are representative of at least two independent experiments.

The incubation of HEK293 cells with ADP‐heptose induces maximal activation of IKKβ after 20 min, which then declines to near basal levels after 60 min [12]. One substrate of IKKβ is the inhibitory IκBα component of NF‐κB. The phosphorylation of IκBα leads to its subsequent Lys48‐linked ubiquitylation by the SCF^βTRCP^ E3 ligase complex, which triggers the proteasomal degradation of IκBα within 30 min (Fig. 4A, lane 2). The degradation of IκBα releases p50, which combines with p65 and translocates to the nucleus where it stimulates transcription of NF‐κB‐dependent genes. Since IκBα is an NF‐κB‐dependent gene, the degradation of IκBα is followed by its rapid re‐synthesis between 60 and 90 min (Fig. 4A, lanes 3–6). We noticed that prolonged stimulation with ADP‐heptose also led to a marked reduction of TIFA expression, which was detectable within 30 min and maximal after about 2 h (Fig. 4A, lanes 1–6). However, in contrast to IκBα degradation, which was blocked by the proteasome inhibitor bortezomib (Fig. 4A, lanes 7–12), the degradation of TIFA was unaffected by this drug (Fig. 4A, lanes 7–12). Instead, the degradation of TIFA was blocked by bafilomycin A1 (Fig. 4A, lanes 13–18), an inhibitor of autophagy, which had no effect on the degradation of IκBα. Thus, IκBα and TIFA are degraded by distinct mechanisms.

Autophagy involves the uptake of a macromolecular cargo from the cytosol into endosomes or autophagosomes, which then fuse with lysosomes, leading to the degradation of the cargo by lysosomal proteases and/or other hydrolases. We observed that the degradation of TIFA was not only blocked by bafilomycin A1 (Fig. 4B, lanes 9–12, uppermost immunoblot) but also by two other inhibitors of autophagy, namely chloroquine and E64‐D (Fig. 4B, lanes 5–8 and 13–16, uppermost immunoblot). Bafilomycin A1 inhibits the vacuolar type H^+^‐ATPase present in lysosomal membranes, preventing the acidification of lysosomes required for lysosomal protease activity [18]. Chloroquine, which is a weak base, may elevate the pH of lysosomes in a non‐specific manner, but has also been reported to inhibit autophagic flux by decreasing autophagosome–lysosome fusion [19]. E64‐D (also known as Aloxistatin) is an irreversible inhibitor of cysteine proteases, including several lysosomal proteases of the cathepsin family [20]. Prolonged exposure of the TIFA immunoblot (Fig. 4B, middle immunoblot) revealed that small amounts of faster migrating species of TIFA were formed during prolonged stimulation with ADP‐heptose, which were not detectable in cells incubated with bafilomycin A1 or chloroquine. They are therefore likely to be fragments of TIFA formed during proteolysis by lysosomal proteases. Consistent with this notion, the most rapidly migrating species were suppressed by E64‐D. In summary, our results establish that ADP‐heptose induces the degradation of TIFA by an autophagic pathway.

### TIFA autophagy requires the expression of TRAF2 or TRAF6

**Fig. 5:**
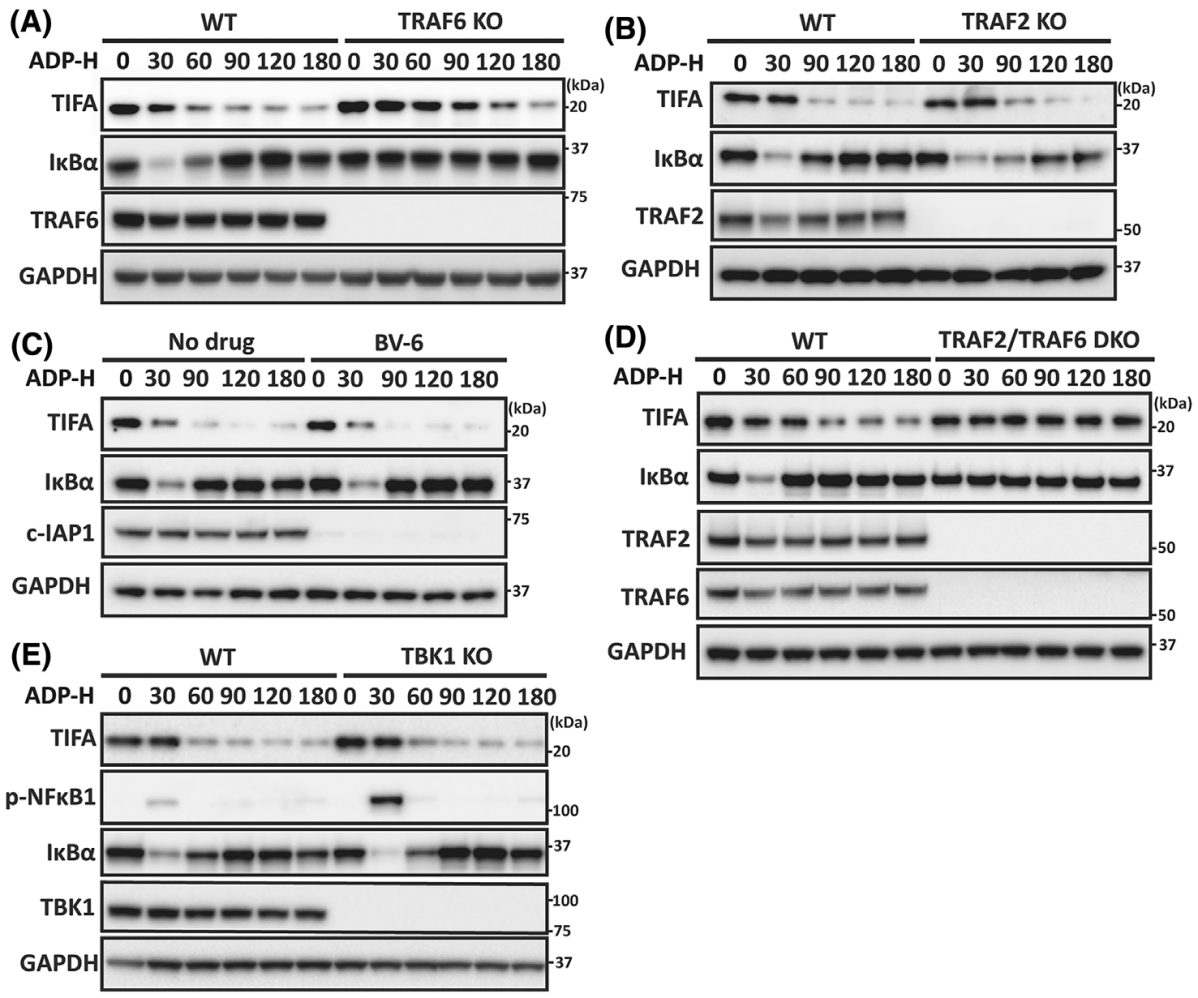
The ADP‐heptose stimulated degradation of TIFA is abolished by the combined deletion of TRAF2 and TRAF6 but not by the individual deletion of TRAF2, TRAF6, cIAP1, or the protein kinase TBK1. (A) Wild‐type (WT) or TRAF6 knockout (KO) HEK293 cells were stimulated with 30 μm ADP‐heptose (ADP‐H) for the times indicated, then lysed and analysed by SDS/PAGE followed by immunoblotting using the antibodies indicated. (B) As in (A), except that wild‐type (WT) and TRAF2 knockout (KO) HEK‐Blue cells were stimulated with 30 μm ADP‐H for the times indicated, then lysed and analysed by SDS/PAGE followed by immunoblotting. (C) As in (A), except that wild‐type (WT) HEK‐blue cells were incubated in the presence or absence of 5 μm BV‐6 for 2 h prior to ADP‐H stimulation. (D) As in (A), except that the degradation of TIFA was compared in WT and TRAF2/TRAF6 double knockout (DKO) HEK293 cells. (E) As in (A), except that ADP‐H signalling was studied in WT and TBK1 KO HEK‐Blue cells. The immunoblots shown are representative of at least two independent experiments.

We next sought to identify the components of the ADP‐heptose signalling pathway required to trigger the degradation of TIFA. Given that TRAF6 expression is essential for the activation of TAK1, the ‘master’ kinase of the signalling pathway [12], we first studied the degradation of TIFA in cells lacking TRAF6. Interestingly, the ADP‐heptose‐dependent degradation of TIFA was still observed in TRAF6 KO cells, although it occurred at a slightly slower rate (Fig. 5A). This finding excluded the possibility that a component of the pathway, ‘downstream’ of TRAF6, was required for TIFA autophagy. We also found that the ADP‐heptose‐dependent degradation of TIFA was unimpaired in either TRAF2 KO cells (Fig. 5B) or in cells treated with BV‐6, a compound that induces the degradation of c‐IAP1 (Fig. 5C). However, TIFA degradation was completely abolished in TRAF2/TRAF6 double KO cells (Fig. 5D), indicating that TRAF2 is required for the ADP‐heptose‐dependent decrease in TIFA expression in TRAF6 KO cells (Fig. 5A) and that TRAF2/c‐IAP1 and TRAF6 operate redundantly to drive TIFA autophagy in wild‐type cells.

Similar to TIFA autophagy, the ADP‐heptose‐stimulated activation of TANK‐binding kinase 1 (TBK1), an IKK‐related kinase, is reduced in TRAF6 KO cells and abolished in TRAF2/TRAF6 double knockout cells [12]. This raised the possibility that TBK1 might be required for TIFA autophagy. However, TIFA degradation was unaffected in TBK1 KO HEK293 cells (Fig. 5E), which excluded this mechanism. Consistent with TBK1 being a negative regulator of the canonical IKK complex [21, 22], the phosphorylation of NF‐κB1 and degradation of IκBα (both dependent on IKKβ activity) were enhanced in TBK1 KO cells (Fig. 5E).

## Discussion

In this paper, we used a deletion/mutagenesis approach in combination with predictions made by alphafold3 to identify how TRAF2 binds to TIFA. We found that a novel motif, Pro‐Xaa‐Xaa‐Glu, between amino acid residues 159 and 162 of TIFA, which has not been identified previously in other TRAF2 binding proteins, was critical for interaction with TRAF2. Glu162 interacts with Lys364 and Trp356 of TRAF2, while Pro159 has an important positioning role. We also found that Gln150 and Glu151 of TIFA comprise a second TRAF2‐interacting motif, that is almost perfectly conserved in vertebrates and interacts with the same amino residues in TRAF2 as the canonical (Pro/Ser/Ala/Thr)‐Xaa‐(Gln/Glu)‐Glu motif present in other TRAF2 binding proteins [14] (Fig. 1). In human TIFA, amino acid 148 is not Pro, Ser, Ala, or Thr but Leu, probably explaining why the Gln‐Glu sequence in TIFA has not been recognised as a TRAF2 binding motif previously. However, although Leu148 is conserved in mammals, it is replaced by Pro in zebrafish (Fig. 3E). Therefore, zebrafish TIFA does have a canonical TRAF2 binding sequence (Pro/Ser/Ala/Thr)‐Xaa‐(Glu/Gln)‐Glu.

The Pro‐Xaa‐Xaa‐Glu motif in TIFA is essential for TRAF2 binding because binding is abolished by the mutation of Pro159 to Gln or Asp, or by the mutation of Glu162 to Gln or Ala in full‐length human TIFA (Fig. 3) and in the *C*‐terminal truncation mutant TIFA[1–157] (Fig. 1), despite the Gln/Glu motif being intact. Nevertheless, Glu151 is also essential because its mutation to Gln or Ala abolishes TIFA binding to TRAF2 (Fig. 3C), even though the Pro‐Xaa‐Xaa‐Glu motif is intact. Thus, both motifs are critical for TRAF2 binding to TIFA during ADP‐heptose signalling.

Although the results presented in this paper establish that TRAF2 and TRAF6 bind to TIFA in entirely different ways, several important questions remain unresolved. For example, how the phosphorylation of intrinsic TIFA dimers induces their oligomerisation and interaction with TRAF2 and TRAF6, and whether TRAF2 and TRAF6 can bind simultaneously to the same TIFA molecule or to different TIFA molecules in TIFAsomes. These issues will only be resolved by more detailed structural analysis.

The alphafold3 model also predicted an interaction between Arg118 of TIFA and Asp445 of TRAF2 and between Tyr21 of TIFA and Glu450 of TRAF2 (Fig. 2). Further research is needed to assess whether these amino acid residues are important for TRAF2 binding. However, Arg118 is replaced by cysteine in the two bird and one reptile sequence shown, suggesting that this residue may not be critical for TRAF2 binding, while Tyr21 is replaced by Phe in the zebrafish (Fig. 3E). Moreover, Tyr21 and Arg118 are in a conserved ordered regions of TIFA close to the FHA domain and may therefore be important for TIFA stability.

In this paper, we have also established that ADP‐heptose induces the degradation of TIFA by an autophagic mechanism that requires the expression of either TRAF6 or TRAF2, but no other component in the signalling pathway that lies ‘downstream’ of TRAF2 and TRAF6, such as the protein kinase TAK1. Ubiquitylation is a recognised signal for the autophagic degradation of protein complexes, whereby autophagy adaptor proteins bind simultaneously to the ubiquitylated cargo and to ATG8 family members present on the forming autophagosome [23]. TRAF6, TRAF2 and c‐IAP1 all undergo ubiquitylation during ADP‐heptose signalling in HEK293 cells catalysed by the E3 ligase activities of TRAF6 and/or c‐IAP1 [12]. It is therefore possible that the roles of TRAF2 and TRAF6 in TIFA autophagy are to undergo autoubiquitylation, permitting the interaction of TIFAsomes with autophagy adaptor proteins to facilitate their uptake into autophagosomes. Further work will be needed to test this hypothesis.

It has been reported that infection of AGS cells with *Helicobacter pylori* induces the degradation of TIFA by both autophagic and proteasomal mechanisms [24]. In the present study, we found that the ADP‐heptose/ALPK1‐dependent degradation of TIFA in HEK293 cells was blocked by inhibitors of autophagy but unaffected by proteasome inhibitors. In the earlier study, the authors reduced the expression of TRAF2 and TRAF6 by incubating AGS cells with siRNA targeting both molecules and reported that this failed to prevent TIFA degradation [24]. In contrast, we found that ADP‐heptose‐dependent TIFA autophagy was abolished in TRAF2/TRAF6 double knockout cells. It is therefore possible that the residual expression of TRAF2 and TRAF6 remaining after siRNA treatment was sufficient to maintain TIFA autophagy. Alternatively, *Helicobacter pylori* might induce degradation by another mechanism that does not require either TRAF2 or TRAF6. Importantly, the finding that the ADP‐heptose‐stimulated autophagic degradation of TIFA occurred within 2 h in our study is consistent with the observation that activation of the cGAS‐STING innate immune pathway induces the autophagic degradation of STING over a similar timeframe [25].

## Author contributions

TS and PC conceptualised the project, designed the experiments and wrote the paper. TS, KH and CT performed experiments, TS and KH prepared figures, TS analysed data and NTW generated reagents.

## Conflict of interest

The authors declare no conflict of interest.

## Peer review

The peer review history for this article is available at https://www.webofscience.com/api/gateway/wos/peer‐review/10.1002/1873‐3468.70110.

## Data Availability

All study data have been included in the main article.
